# Early, but not late visual distractors affect movement synchronization to a temporal-spatial visual cue

**DOI:** 10.3389/fpsyg.2015.00866

**Published:** 2015-06-24

**Authors:** Ashley J. Booth, Mark T. Elliott

**Affiliations:** ^1^School of Psychology, University of Birmingham, Edgbaston, UK; ^2^Institute of Digital Healthcare, Warwick Manufacturing Group, University of Warwick, Coventry, UK

**Keywords:** sensorimotor synchronization, visual cues, movement timing, distractor cues

## Abstract

The ease of synchronizing movements to a rhythmic cue is dependent on the modality of the cue presentation: timing accuracy is much higher when synchronizing with discrete auditory rhythms than an equivalent visual stimulus presented through flashes. However, timing accuracy is improved if the visual cue presents spatial as well as temporal information (e.g., a dot following an oscillatory trajectory). Similarly, when synchronizing with an auditory target metronome in the presence of a second visual distracting metronome, the distraction is stronger when the visual cue contains spatial-temporal information rather than temporal only. The present study investigates individuals’ ability to synchronize movements to a temporal-spatial visual cue in the presence of same-modality temporal-spatial distractors. Moreover, we investigated how increasing the number of distractor stimuli impacted on maintaining synchrony with the target cue. Participants made oscillatory vertical arm movements in time with a vertically oscillating white target dot centered on a large projection screen. The target dot was surrounded by 2, 8, or 14 distractor dots, which had an identical trajectory to the target but at a phase lead or lag of 0, 100, or 200 ms. We found participants’ timing performance was only affected in the phase-lead conditions and when there were large numbers of distractors present (8 and 14). This asymmetry suggests participants still rely on salient events in the stimulus trajectory to synchronize movements. Subsequently, distractions occurring in the window of attention surrounding those events have the maximum impact on timing performance.

## Introduction

Nodding or tapping along to a favorite song is often something we do with little conscious thought. This demonstrates the automaticity of being able to move in time to a rhythmic stimulus, an ability that forms the basis of sensorimotor synchronization (SMS) research (Repp and Su, 2013). The majority of SMS research has focussed on the timing of movements to an auditory rhythmic cue and indeed it appears this is the sensory modality that facilitates the most accurate timing of movements (Repp and Penel, 2004; Elliott et al., 2010). However, movement synchrony can also occur outside the context of music. In social situations, groups of individuals can spontaneously coordinate the timing of their movements, for example, two people falling into step when walking together (Zivotofsky et al., 2012), or an excited crowd bouncing up and down together in a sports stadium (Noormohammadi et al., 2011). In these group scenarios, visual cues are likely to provide a strong timing stimulus that results in implicit synchrony emerging within the group. However, with each person in a group exhibiting slightly different timing properties, it is currently unclear how synchrony occurs in the face of conflicting visual cues. Here, we have developed an experimental paradigm that investigates how individuals synchronize movements to a target visual cue in the presence of conflicting visual stimuli.

Timing accuracy in SMS studies is often quantified by the *asynchronies*, which represent the time difference between the target and the executed movement. The mean and variability of the asynchronies are taken into account. A negative mean asynchrony (NMA) is usually observed in SMS research where the movement typically precedes the target by 30–50 ms (Aschersleben and Prinz, 1995). While auditory cues dominate SMS research, other modalities have been investigated. In particular, SMS to a discrete flashing visual stimulus results in reduced timing accuracy in terms of asynchrony variability (Repp and Penel, 2004; Kurgansky, 2008; Elliott et al., 2010; Wright and Elliott, 2014) compared to an auditory metronome. Hence, discrete auditory stimuli provide a more reliable, salient cue compared to a discrete rhythmic visual cue (Repp and Penel, 2004). However, more recent studies found that synchronizing movement to continuous visual cues, i.e., those exhibiting temporal and spatial information, yielded strong SMS that was comparable to studies using auditory cues (Hove et al., 2012; Varlet et al., 2012; Armstrong et al., 2013). Moreover, visual trajectories representing biologically compatible movements further facilitates rhythm perception (Su, 2014a) and movement synchronization (Su, 2014c). This latter finding indicates how the temporal-spatial visual information provided by surrounding members of a group could influence the implicit synchrony of movements within the group.

A number of studies have implemented a distractor paradigm to observe how irrelevant cues presented in auditory or auditory versus visual modalities can affect an individual’s ability to synchronize their movements to a target cue. As might be expected, an auditory distractor in the presence of a discrete visual target leads to a strong distraction effect, due to the strong saliency of the auditory modality (Repp and Penel, 2002, 2004). These distraction effects are quantified through a change in NMA, i.e., asynchronies becoming more negative in the presence of early distractors or more positive for late distractors, and asynchrony variability, with strong distractor effects reducing the stability of the asynchronies. In general, discrete distractor cues (be it auditory–auditory or auditory-visual modalities) exhibit an asymmetric NMA effect, where a strong attraction is observed when the distractor precedes the target, but show little change for late distractors (Repp, 2003; Repp and Penel, 2004).

What is currently unclear is how an individual’s ability to synchronize movements to temporal-spatial visual cues is affected by similar conflicting visual distractors. In this study, we investigated participants’ ability to synchronize oscillatory arm movements in time to a temporal-spatial oscillating visual target, in the presence of identical visual distractors offset in phase to the target. As well as varying phase to influence the temporal relation between target and distractor, we also varied the visual impact of the distraction effect by varying the number of distractor stimuli present. Increasing the number of distraction stimuli should correspondingly increase visual attention to the distractors (Bartram et al., 2003). Hence we predicted that the strength of the distraction effect would be a function of both the temporal separation and the number of distractors present. As observed in previous studies, we further expected that the temporal distraction would be at it’s greatest when the phase offset was around a quarter of the oscillation period (Repp, 2003; Repp and Penel, 2004). However, due to the continuous nature of both the movements and the stimuli, we did not expect to see an asymmetry in the distraction effect as observed with discrete stimuli paradigms.

## Materials and Methods

### Participants

Eleven University of Birmingham undergraduate Psychology students (six female; *M*_age_ = 18.4, range = 18–20, SD 0.67 years) gave written informed consent to take part in the study. All participants reported themselves free of any neurological disease, head trauma, musculoskeletal impairment, visual impairment, or hearing impairment. Ethical approval was granted by the University of Birmingham Science, Technology, Engineering, and Mathematics Ethical Review Committee. Of the 11 participants, nine were right-handed. Data from one participant was removed due to difficulty with following instructions and completing the task correctly.

### Experimental Setup

**FIGURE 1:**
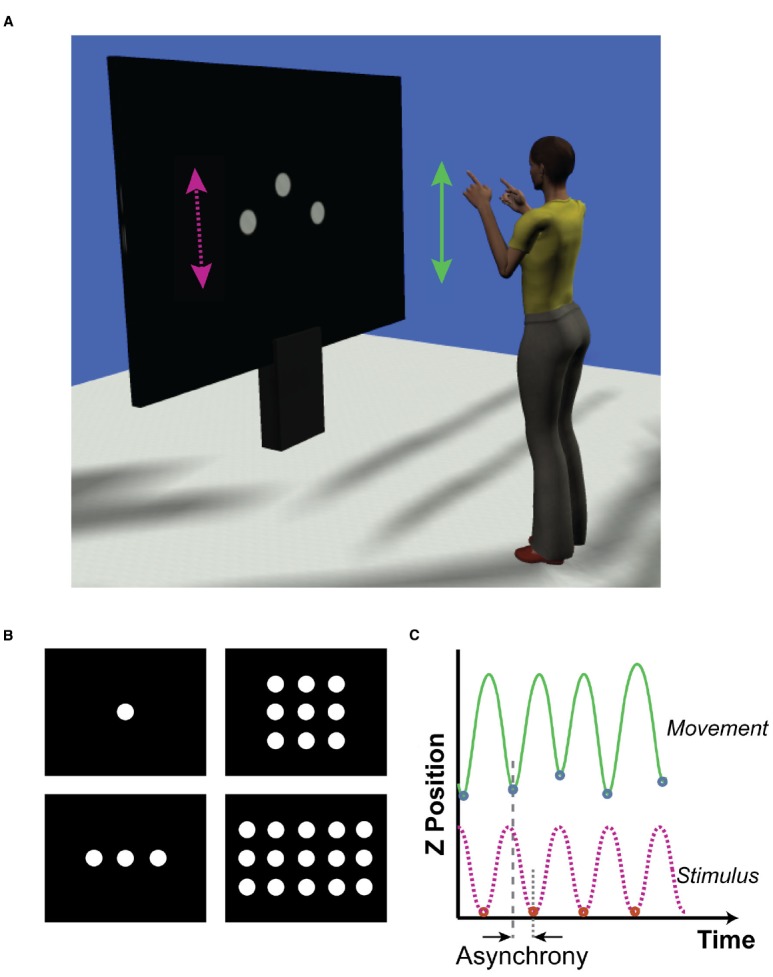
**(A)** Representation of experimental set up. Participants faced a large projection screen which presented the visual stimuli. The stimuli (100 pixel diameter dots) moved vertically up and down, following a sinusoidal trajectory. The target stimulus was always the center dot. Distractor dots moved out of phase with the target by 0, ±100, ±200 ms. Participants made bimanual arm movements in synchrony with the target stimulus, flexing and extending the forearm from the elbow. **(B)** Formation of target and distractor stimuli. We investigated if the distraction effect was a function of the number of distractor stimuli. The number of distractor stimuli was varied across trials such that there were no distractors (top left), two distractors (bottom left), eight distractors (top right), or 14 distractors (bottom right). **(C)** Measurements of timing accuracy. Representative trajectories of the target stimulus (bottom trace, dashed pink) and the corresponding participant’s dominant arm movement (top, solid green) are shown. We extracted the times of the minimum positions for each movement oscillation along with the times of the minimum stimulus positions. Asynchrony was calculated by subtracting the time of movement event from the time of the corresponding stimulus event.

Participants stood on a marked point 1.85 m from a projection screen (1.6 m wide × 1.2 m tall; Figure 1A). Arm movement trajectories were captured using a 12-camera Qualisys Oqus motion capture system (Qualisys AB, Gothenburg, Sweden), with adhesive reflective markers attached to the shoulders, elbows, wrists, and index fingers of both arms. The camera system operated with a sampling rate of 200 Hz.

### Stimuli

Visual stimuli were generated in Matlab (2013a; The Mathworks, MA, USA) Psychophysics Toolbox (Brainard, 1997). The stimuli consisted of a series of white circular dots (100 pixels diameter) moving vertically against a black background with a sinusoidal trajectory (period: 800 ms, 60 frames per second). The peak–peak range of movement for the dots was 200 pixels. Participants were instructed to synchronize movements with the “target”—a centrally positioned dot that was present in all conditions. In addition, a number of distractor dots were positioned symmetrically to the sides, above and below the target. There were four distractor conditions, which consisted of 0, 2, 8, or 14 distractor dots in the formations shown in Figure 1B. Dots were separated from one another, center to center, by 125 pixels horizontally and 200 pixels vertically. In addition to the different numbers of distractors, there were five “phase-offset” conditions where the timing of the distractor dots was offset such there was a constant phase lead (negative) or lag (positive) of 0, ±100, or ±200 ms relative to the central target trajectory. The spacing of the dots was designed such that none of the phase-offset conditions resulted in occlusion of the target dot on the screen. A digital high (+5V) signal pulse was output via a data acquisition card (USB-6343, National Instruments, TX, USA) to the Qualisys motion capture system each time the target dot reached its minimum position in the trajectory. This was used to align screen output with the participant’s movements (see Data Processing).

### Experimental Design and Procedure

Participants completed the study individually. They were instructed to move both forearms up and down in synchrony with the central target dot, flexing and extending at the elbows with only their index fingers extended. We instructed the use of bimanual movements to improve timing stability (Helmuth and Ivry, 1996). In addition, during pilot tests participants reported bimanual movements to be more comfortable and natural for the task. Participants were further required to keep their wrists tense and so were instructed to keep their wrists firm such that a straight line could be imagined between the fingertip and elbow during the movement. They were told to ignore the movements of the non-target dots to the best of their ability. A practice trial was carried out to ensure that the requirements were fully understood and they were ready to continue.

There were three trials for each condition (3 Distractors conditions: 2, 8, 14 × 5 Phase offset conditions: –200, –100, 0, 100, 200 ms; plus a no-distractor condition) totalling 48 trials in all. The order of the trials was randomized for each participant to avoid order effects. Each trial lasted 40 s, which resulted in 50 dot oscillations per trial.

### Data Processing

Only the vertical (z-axis) data from the reflective marker attached to the index finger on the dominant hand was used for analysis. Using a peak detection algorithm from the MatTAP toolbox (Elliott et al., 2009b), the “event times” of the lowest vertical points of the executed oscillatory arm movements were extracted (Figure 1C). Lowest points were chosen as evidence suggests synchronization is more stable on the downward movement (Miura et al., 2011). Similarly, the event times of the lowest positions of the target stimulus were recorded as the time at which the digital signal from data acquisition was set high (see Stimuli). The first five event times from each trial were discarded from the analysis to allow for participants to initially synchronize with the target. The event times between the stimulus and the participant’s movements were then aligned (Elliott et al., 2009b) by finding the movement onset time closest to each stimulus onset time (on average <1% of all stimulus onsets could not be aligned to a participant’s corresponding movement, indicating participants were able to perform the task). Subsequently, the asynchronies were calculated as the time difference between the stimulus event and the corresponding movement event. A negative asynchrony indicated that the movement event occurred before the stimulus (Figure 1C).

The standard deviation and mean asynchrony were calculated for each trial and the average taken across trials for each participant. We initially analyzed the effects of the number of distractors and phase offset (reported in sections “Mean Asynchrony” and “Standard Deviation”) using a 3 (Distractors: 2, 8, 14) × 5 (Phase Offset: –200, –100, 0, 100, 200 ms) repeated measures design. We further analyzed just the effect of number of Distractors using data from the 0 phase-offset conditions in addition to the baseline “no distractor” condition [4 (Distractors: 0, 2, 8, 14) × 1 (Phase Offset: 0 ms) repeated measures; reported in section “Comparison of No-Distractor with Distractor Conditions”]. Statistical analysis was completed using Repeated Measures ANOVAs in SPSS (version 21, IBM Corp., NY, USA). Significance levels were set to *p* < 0.05. Greenhouse–Geisser adjustments were made for results that violated sphericity assumptions. *Post hoc* analyses were adjusted for multiple comparisons using the Bonferroni method.

## Results

### Mean Asynchrony

**FIGURE 2:**
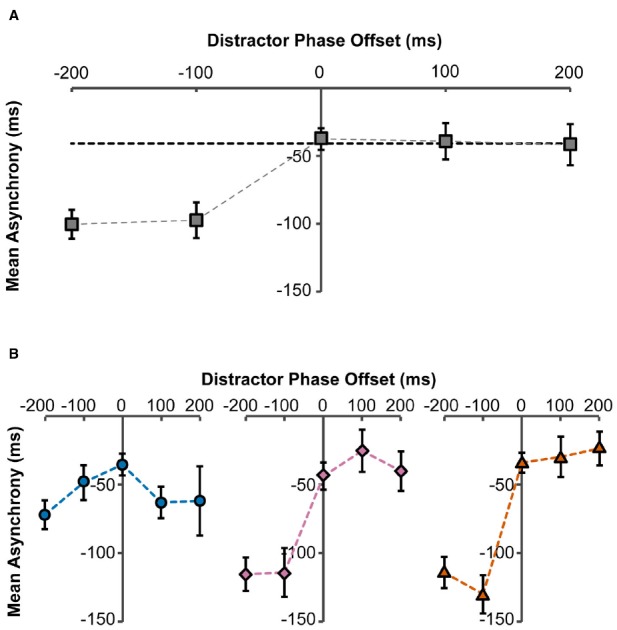
**Mean asynchrony as a function of distractor phase-offset to target stimulus.** Asynchronies were measured between the participant’s movements and the target stimulus. **(A)** Overall effect of distractor phase-offset on mean asynchrony, collapsed across number of distractors. Error bars represent standard errors. Dashed horizontal black line indicates baseline mean asynchrony in the no-distractor condition. **(B)** Effect of distractor phase-offset on mean asynchrony, with two distractors present (circles), eight distractors present (diamonds), and 14 distractors present (triangles). Error bars represent standard errors.

A repeated measures within-participants ANOVA revealed that there was a significant effect of phase-offset on mean asynchrony [*F*(4,36) = 25.17, *p* < 0.001]. That is, changes to the phase-offset significantly affected synchronization to the target (Figure 2A). *Post hoc* analysis identified that there were only significant differences between the 0 ms phase-offset condition relative to the –200 ms condition (*M* = –62.8 ms, *p* < 0.001) and the –100 ms condition (*M* = –59.8 ms, *p* = 0.001). However, there were no significant differences between the –200 ms and –100 ms phase-offsets conditions, and so performance does not continue to decline linearly as the phase-offset increases. Moreover, the positive phase offsets did not significantly alter the mean asynchrony compared to the 0 ms phase-offset. These findings show that there is an asymmetrical effect of phase-offset where the negative phase-offset conditions make the mean asynchrony more negative, so arm movements were drawn to the phase-leading distractor trajectories. In contrast, movements were not drawn to phase-lagging distractor trajectories.

There was no significant main effect of the number of distractors on the mean asynchrony; however, the analysis yielded a significant interaction between the number of distractors and phase-offset [*F*(2.7,24.4) = 13.36, *p* < 0.001; Figure 2B]. Analyzing each Distractor condition separately highlighted that when only two distractors were present, there was no effect of phase-offset on the mean asynchrony [*F*(1.58,14.26) = 1.83, *p* = 0.199]. In contrast, for the 8 dot [*F*(4,36) = 32.79, *p* < 0.001] and 14 dot [*F*(4,36) = 36.48, *p* < 0.001] distractor conditions, the previously described phase attraction for leading distractors was present (Figure 2B).

### Standard Deviation

**FIGURE 3:**
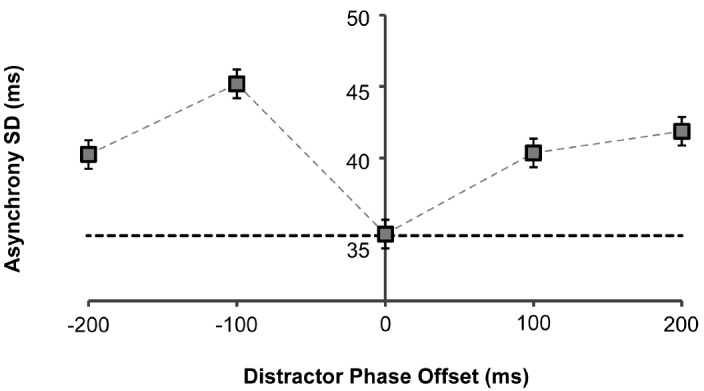
**Asynchrony standard deviation (SD) as a function of distractor phase-offset to target stimulus.** There was no significant effect of number of distractors on the asynchrony SD, so results are collapsed across this condition. Error bars represent standard errors. Dashed horizontal black line indicates baseline mean asynchrony in the no-distractor condition.

We further investigated how the distractors impacted on the variability (standard deviation) of the asynchronies over a trial. Again, we observed a significant main effect of phase-offset [*F*(4,36) = 5.14, *p* = 0.002; Figure 3]. *Post hoc* analyses identified the –100 ms phase-offset as the only condition that significantly differed from the 0 ms phase-offset condition (*M* difference = 10.5, *p* = 0.014). We found that in this condition, the variability of asynchronies significantly increased, indicating that the strongest distraction occurred when the distractor stimuli were moving earlier in phase by around 100 ms.

### Comparison of No-Distractor with Distractor Conditions

Two further analyses were carried out to compare a no-distractor condition (i.e., only the target stimulus present) with the other multiple dot conditions where there was no phase-offset between the target and distractor stimuli. As expected, we found no significant effect of the number of distractors on the mean asynchrony (*p* = 0.089) or the standard deviation (*p* = 0.765). Hence we can conclude that the number of distractors alone does not significantly affect synchronization to a target visual cue where there is no phase-offset applied.

## Discussion

In this study, we investigated how we synchronize our movements in time with a visually oscillating target cue in the presence of same-modality distractor cues. Participants were instructed to synchronize oscillatory arm movements in time with the target cue, while distractors varied in phase (either lagging or leading the target cue) and number. We found that, as predicted, the degree of phase-offset between distractor and target stimuli significantly affected the asynchrony of the participants’ movements to the target cue. However, contrary to expectations, an asymmetry in the distraction effect was observed, with only phase-leading distractors (–100, –200 ms) influencing the asynchrony; lagging distractors did not show any significant effect on performance. In particular, a phase offset of –100 ms appeared to have a substantial impact on performance, both in terms of greater negative asynchrony and higher asynchrony variability. We further found the distraction only occurred with larger numbers of distractor stimuli surrounding the target; we saw no effect when there were only two distractor stimuli present.

The effect of distractor cues on sensorimotor synchronization performance has been investigated for combinations of auditory–auditory (Repp, 2003), auditory-visual (Repp and Penel, 2004; Hove et al., 2012; Debats et al., 2013) and auditory-proprioceptive cues (Debats et al., 2013). An asymmetry in the strength of the distraction has been observed in auditory–auditory and auditory-visual conditions (Repp, 2003; Repp and Penel, 2004). In both cases, a strong influence of the auditory distractors on the asynchronies was observed when the distractors occurred earlier than the target cue, but not later. With discrete cues, this is expected: the participant’s attention is captured by the early distraction events and hence draws the motor responses away from the target cue. Later distraction cues are less likely to capture attention as they occur after the motor action has been planned and executed (Repp, 2003). With a continuously present visual cue and continuous motor action however, we expected there to be no difference between a distractor being late or early in phase. We considered that the continuous signal would be a constant distraction and hence would show a symmetrical effect on the asynchronies regardless of them leading or lagging the target. The fact that we saw an asymmetry indicates that participants were still utilizing salient points in the sensory stimuli and aligning them to similarly salient anchor points in their own movement trajectories. For the visual cues, the salient points could have been, for example, the change in direction of the moving dot at the top or bottom of the sinusoidal trajectory. Indeed, it makes sense to have discrete points of reference for synchronization. On the one hand it has been shown that synchronization to continuous temporal-spatial visual cues is much easier and results in enhanced synchrony performance (Varlet et al., 2012; Armstrong et al., 2013; Armstrong and Issartel, 2014) compared to the task of timing movements to discrete visual cues (Repp and Penel, 2004; Elliott et al., 2010; Wright and Elliott, 2014). However, while the dynamic spatial element of visual information is clearly important for anticipatory timing, it would be inefficient to continuously align and correct movements at arbitrary points in the cue trajectory, just because there is the sensory information available. Evidence from this experiment and other studies (Luck and Sloboda, 2008; Hajnal et al., 2009; Su, 2014b; Varlet et al., 2014) suggests that if we’re timing movements to an external cue, we pick out discrete salient points for temporal alignment that allows us to efficiently correct movements through each repetition of the cycle. These do not have to be explicit observable events within the trajectory but can be related to derivatives of the movement such as velocity (Su, 2014b; Varlet et al., 2014) or peak acceleration (Luck and Sloboda, 2008). Similar strategies arise in the movements themselves. Producing smooth continuous movements results in the timing emerging from the movement itself [referred to as emergent, or implicit timing (Spencer et al., 2003)]. This smooth movement reduces the ability to make accurate corrections necessary for maintaining synchrony (Elliott et al., 2009a). Hence it is beneficial to timing performance to have relatively discrete (identified by a high level of jerk) features in the movement that allows event based or explicit timing (Elliott et al., 2009a). Again, in this case it is likely that proprioceptive feedback of the change of direction at the lowest point of the movement was sufficient to allow participants to synchronize their actions. These strategies of extracting discrete timing events from continuous cues and movements explains why we see a similar asymmetrical distraction effect in this task as in previous experiments that used discrete cues (e.g., an auditory metronome) and movements (finger tapping).

To understand the effect of the distractors further, we must consider the underlying attentional processes. Moving visual stimuli in the periphery attracts attention far better than static stimuli (Bartram et al., 2003). In addition, jerky motion captures attention more than smooth motion (Sunny and von Mühlenen, 2011). Our study shows that even if visual stimuli are not being attended to, the salient features of the distractor cue trajectory attract coordinated movements away from a target stimulus. It appears however, that the number of distractors and possibly the spatial location is also important. We only observed the strong distraction effect when there were 8 or 14 distractors present. This is likely to be due to the increased salience of the distractor cues, with the large number of stimuli moving at the same phase making them increasingly difficult to ignore (Bartram et al., 2003). Equally, the salience could have been increased by the larger number of distractors completely surrounding the target dot, rather just on either side, as in the two-distractor condition. Our results therefore suggest a bottom-up stimulus driven attentional process is in place (Theeuwes et al., 2000) where the saliency of the distractor relative to the target is what draws the attention of the individual. The temporal distance of the distractors from the target is a further important factor in the strength of the distraction. With the peak distraction effect occurring when the distractors are –100 ms earlier than the target cue, it is likely a temporal window of attention (Naccache et al., 2002) around the salient event in the target cue is present. If the distractor cue event falls into this window, then it maximizes attentional capture from the target (due to the multiple distractors providing a stronger stimulus than the target). This is somewhat different to the well-documented “window of integration.” Sensory integration of temporal cues occurs when two stimuli are deemed relevant to one-another and occur close together in time (Elliott et al., 2014). In this scenario, the stimuli are integrated in a fashion that can be described under a Bayesian framework, such that the resulting combined cue becomes more reliable than either of the individual stimuli (Ernst and Banks, 2002). In a synchronization task this results in a reduced variability of the timed movements (Elliott et al., 2010). Here, we explicitly inform participants to ignore the distractor stimuli, so they are aware they are not relevant to the target. Subsequently, we observe a high level of variability at the –100 ms offset, which is likely due to be a result of the conflict between the top-down goal of synchronizing with the target cue and the bottom-up stimulus driven effect of being attracted to the more salient distractor stimuli.

Finally, we consider these results in the context of interpersonal synchrony. Spontaneous synchrony can emerge between two individuals, often due to the strong visual cues from the partner (Richardson et al., 2007; Zivotofsky et al., 2012). Considering larger groups (e.g., crowds jumping up and down in a sports stadium), there is potentially a contextual effect on how synchrony may emerge within a group. On the one hand, an individual may be focussed on timing their movements with a known partner, in which case the movements of the remaining crowd act as distractors and hence, based on our results, are likely to weaken the coupling between the dyad. Alternatively, an individual may be moving as part of the larger crowd, in which case it would be advantageous to combine the cues from all surrounding individuals. Through sensory integration, this latter scenario should result in greater stability of synchrony within the group. In reality, a combination of these processes are likely to be present, such that within a crowd we observe an overall weak coupling across all individuals, but with strong synchrony couplings between small numbers of individuals within the crowd.

### Conflict of Interest Statement

The authors declare that the research was conducted in the absence of any commercial or financial relationships that could be construed as a potential conflict of interest.
